# Integrating tuberculosis and HIV services for people living with HIV: Costs of the Zambian ProTEST Initiative

**DOI:** 10.1186/1478-7547-6-2

**Published:** 2008-01-23

**Authors:** Fern Terris-Prestholt, Lilani Kumaranayake, Rokaya Ginwalla, Helen Ayles, Ignatius Kayawe, Mary Hillery, Peter Godfrey-Faussett

**Affiliations:** 1 London School of Hygiene and Tropical Medicine, Keppel Street, London WC1E 7HT, UK; 2 Zambart Project, UTH School of Medicine, PO Box 50110, Lusaka, Zambia; 3 (previous address) Kara Counselling and Training Trust (KCTT), PO Box 37559, Lusaka, Zambia; 4 Community Home Based Care Programme, Archdiocese of Lusaka, PO Box 32754, Lusaka 10101, Zambia

## Abstract

**Background:**

In the face of the dual TB/HIV epidemic, the ProTEST Initiative was one of the first to demonstrate the feasibility of providing collaborative TB/HIV care for people living with HIV (PLWH) in poor settings. The ProTEST Initiative facilitated collaboration between service providers. Voluntary counselling and testing (VCT) acted as the entry point for services including TB screening and preventive therapy, clinical treatment for HIV-related disease, and home-based care (HBC), and a hospice. This paper estimates the costs of the ProTEST Initiative in two sites in urban Zambia, prior to the introduction of anti-retroviral therapy.

**Methods:**

Annual financial and economic providers costs and output measures were collected in 2000–2001. Estimates are made of total costs for each component and average costs per: person reached by ProTEST; VCT pre-test counselled, tested and completed; isoniazid preventive therapy started and completed; clinic visit; HBC patient; and hospice admission and bednight.

**Results:**

Annual core ProTEST costs were (in 2007 US dollars) $84,213 in Chawama and $31,053 in Matero. The cost of coordination was 4%–5% of total site costs ($1–$6 per person reached). The largest cost component in Chawama was voluntary counselling and testing (56%) and the clinic in Matero (50%), where VCT clients had higher HIV-prevalences and more advanced HIV. Average costs were lower for all components in the larger site. The cost per HBC patient was $149, and per hospice bednight was $24.

**Conclusion:**

This study shows that coordinating an integrated and comprehensive package of services for PLWH is relatively inexpensive. The lessons learnt in this study are still applicable today in the era of ART, as these services must still be provided as part of the continuum of care for people living with HIV.

## Background

Recently there have been major improvements in access to treatment for people living with HIV (PLWH) in developing countries [1]. Provision of anti-retroviral therapy (ART) has moved to the top of the international health policy agenda and scaling-up is in rapid progress. However, ART is only one of many services critical to PLWH throughout their lives. It is widely acknowledged that to make a dent in the HIV epidemic in Africa an integrated approach is needed, addressing prevention, treatment and care [1-4]. The Zambian ProTEST Initiative was a pilot study of an integrated model for providing a comprehensive package of services for PLWH at all stages of infection. This package was developed prior to the availability of ART, but remain important in the current service package for the continuum of care. Services within this package include voluntary counselling and HIV testing (VCT) with outreach by youth friendly services, active TB case-finding, TB preventive therapy, an HIV-clinic, HBC, and a hospice, each within a coordinated network of service providers and referral systems.

The appropriateness of these individual ProTEST components has previously been established. Co-ordination of services is expected to improve access to prevention and care [5,6]. VCT has been shown to induce short-term reported behaviour change, that should reduce the incidence of HIV [7-10]. VCT also provides a valuable opportunity for active TB case finding, for referral to and/or provision of TB treatment and prevention, and for referral to other services for PLWH [11]. Isoniazid preventive therapy (IPT) reduces the incidence of TB in PLWH [12-16]. Both screening, treatment and IPT may lead to the prevention of secondary TB cases, by shortening and preventing the infectious period of TB in HIV co-infected people [5]. HBC can alleviate the pressure on hospital in-patient services, lighten the load on family caregivers, improve quality of life for AIDS patients and reduce stigma related to HIV/AIDS [17-22] in [23,24]. It is likely that this would also hold for palliative care and hospice care. Moreover, palliative care that treats the side effects of ART will improve adherence to ART [25-27]. Providing a range of services for PLWH, such as IPT, ProTEST clinic, hospice and HBC, is also likely to serve as an incentive to present earlier for HIV testing [5].

Although some evidence exists on the costs of these individual services (VCT, IPT, HBC) [28-37], there is little data on the costs of providing services jointly and no costs of hospice care in Africa have been published. This study examines the costs of both starting-up and implementing a package of HIV services in Lusaka, Zambia. These provide baseline cost estimates that have been identified as lacking in many estimates of global resources needed for providing a full continuum of care for PLWH [38].

## Methods

### Study sites

The Zambian ProTEST pilot project began in 1999 as a collaboration including the Zambian AIDS-related TB project (ZAMBART), Kara Counselling and Training Trust (KCTT), local primary health clinics, youth friendly services (YFS), district health authorities, and the Archdiocese of Lusaka. Together they provided: VCT with IPT for TB prevention, community outreach, a clinic for management of HIV-related illness, hospice, and HBC. Although these services operated independently, through collaboration and improved referral systems they formed an integrated package. The initial impetus for the intervention was the dual HIV/TB epidemic being seen in Lusaka with its high HIV prevalence (22%) [39] and high incidences of TB (653/100,000 per annum) in Zambia in 2001 [40]. Public sector ART was introduced as a pilot in 2002, and was rapidly rolled out and scaled up [41]. By March 2007, 98,500 people were receiving ART in Zambia [42]. This is about half of the estimated 200,000 people in need of ART [42]. Improved referral systems need to be in place to support this rapid scale up of ART underway in Zambia.

ProTEST Zambia was implemented in two high-density low income urban compounds in Lusaka: Chawama and Matero. Prior to ProTEST, the Chawama health clinic already had a VCT centre providing IPT as part of a study. The ProTEST Initiative in Chawama built a new building for this growing service, introduced monthly co-ordination meetings and strengthened the referral system, supported YFS to provide outreach, and established the ProTEST clinic. The costing took place in the second year of ProTEST operation. Matero was a completely new site, opening in November 2000. ProTEST established a VCT and other ProTEST services, and included chest x-rays as part of their process for TB screening. The two sites differed slightly in their spatial integration: Chawama's VCT and clinic were in stand-alone buildings within the PHC grounds, while in Matero they were situated within the main hospital building.

### Intervention components

The core components of ProTEST were considered to be co-ordination, VCT, IPT, ProTEST clinic, and outreach. Hospice and HBC were considered affiliated services. The heart of the ProTEST Initiative was coordination of the different services. This was done by the ProTEST team at ZAMBART. The team consisted of a medical doctor/manager, a clinical officer, an outreach worker/drama coordinator, a driver, and had input from a secretary (50%) and an expatriate director (20%). The team also participated directly in many of the services provided. They set up and monitored the IPT, ran the weekly ProTEST clinics, and organised the outreach programme. The clinicians provided weekly medical services to the hospice.

#### Coordination

**Figure 1:**
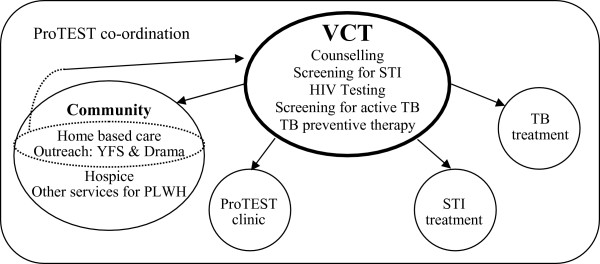
**ProTEST Zambia and its referral systems**. Arrows depict the direction of referrals; VCT: Voluntary counselling and testing; YFS: Youth friendly services; PLWH: People living with HIV; STI: sexually transmitted infection.

The aim of the coordination component was to strengthen collaboration and channels of communication between traditionally vertical TB and HIV services that were serving much of the same population. The main tangible activity was meetings among the different service providers to coordinate their services and strengthen their mutual referral systems (Figure 1). Lunch and transportation were provided to participants. Service providers attending meetings included representatives from all core and affiliated services and other groups working on HIV or TB such as support groups for PLWH, government department of health employees, and other community organisations.

In Chawama, meetings ranged from 12 to 40 participants, averaging 33 participants, and in Matero they ranged from 15 to 25 participants, averaging 19 participants. Although the meetings were planned as monthly events, during the period of the costing, there were 6 meetings in Chawama. In Matero there were 5 meetings during the 3-month start-up period and 3 in the year of the costing.

#### VCT and IPT

VCT served as the entry point for the ProTEST Initiative. Services were provided by nurses with counselling training. During pre-test counselling clients were syndromically screened for STIs, and if need be referred for treatment. Rapid HIV tests were used, making it possible to provide test results to clients on the same day. During post-test counselling HIV-infected people were screened for active TB. People with suspected active TB were referred to the ProTEST clinic for further evaluation of TB and if confirmed they were sent to the national TB services. All other HIV-positive clients with no TB symptoms were offered IPT. IPT consisted of a six-month self-administered course of isoniazid (300 mg daily), collected monthly by clients from the VCT counsellors. Additionally, VCT clients were provided information on services for PLWH.

The Chawama VCT centre was located in a stand alone building in the hospital compound. It had a large reception with smaller counselling rooms around the edge. During the year of the costing counsellors increased from three to six. In addition to the outreach activities described below, the Chawama VCT centre used community mobilisers to draw people in for VCT, who were provided a bonus for each person presenting for pre-test counselling. In Matero, the VCT was a room within the main hospital building. There was a single counsellor. The waiting room was a bench in the hospital corridor.

#### Outreach

Outreach was provided through YFS. With support of the ZAMBART drama coordinator, YFS volunteers wrote and performed dramas in the communities and recorded them on video to provide information on HIV prevention, the ProTEST Initiative, and the benefits of VCT. Each group received a nominal allowance. A 4-month outreach project targeted at sex-workers and their clients was implemented by the Chawama YFS. YFS in Chawama was allocated two rooms in the new VCT building. In Matero the health clinic provided them with a room.

#### ProTEST clinic

The ProTEST clinics served HIV-infected individuals who had passed through VCT. These weekly clinics specialised in treating opportunistic infections and other HIV-related diseases and they were staffed by the ZAMBART doctor and medical officer. The clinicians also referred clients to community services such as hospice and HBC. Drugs were provided free of charge to patients. Treatment did not include the provision of ART during the time of this study, although ProTEST clients have more recently been able to access ART. The ProTEST clinic was held in 2 rooms of the newly built ProTEST building in Chawama, and in a consulting room in the hospital in Matero.

#### HBC

The Archdiocese of Lusaka ran a community HBC programme throughout Zambia, including Chawama and Matero. Volunteer caregivers provided care and delivered food supplements to ill patients in their homes. With their strong links in the communities, they were in a position to refer people for VCT and into the ProTEST system.

#### Hospice

In addition to VCT, KCTT also ran a hospice which served terminally ill patients referred in from Chawama, Matero, and other compounds. The hospice had a total of 20 beds for adults. In addition to core staff, ZAMBART provided the services of a medical doctor and a medical officer to the hospice on a weekly basis. Community organisations provided about a third of the caregiver time.

### Costs

Cost data collection was based on the *Costing Guidelines for HIV Prevention Strategies *[43]. In both sites annual costs and intermediate outcome indicators were collected between November 2000 and October 2001, with the exception of HBC where the period was January to December 2000.

Full costs were collected for all ProTEST components, except IPT, where incremental costs of adding IPT on to routine VCT services were estimated. Full costs include the value of space, vehicles, and all operating expenses. In Matero, this also includes a depreciated value of start-up costs.

All costs were adjusted to 2007 US dollars ($), including costs quoted from other studies, using a GDP deflator [44], the 2006 GDP deflator (3.2%) was also applied to 2007 as this was not yet available. The average exchange rate during the main costing period was used (3,622 Kwacha per US Dollar ($)) [45].

Financial and economic costs were estimated from a provider's perspective. Financial costs represent actual expenditure on goods and services purchased. These were based on financial accounts and financial reports. Economic costs include the estimated value of all resources used in the intervention, such as the opportunity cost of service providers attending coordination meetings, YFS and HBC volunteers, and donated goods such as HIV test kits and space. An inventory of donated time and volunteer staff input was made either from logs of donations or interviews during site visits. Unit costs were assessed for volunteer time by either the salary of the individual donating time or minimum wage, for unemployed individuals. The value of donated goods were assessed from market prices (for food donations), pharmacies (for medicines and medical supplies) and from the internet (for HIV test kits not available in country). The annual financial cost of capital goods was calculated using straight-line depreciation, in which the total cost of the good was divided by the length of its working life. The annual economic cost of capital goods was calculated using a 3% discount rate, as recommended by Weinstein et al. [46].

Cost data were collected from administrative records from the service providers (KCTT, ZAMBART, and the Archdiocese of Lusaka), observation at study sites, and discussions with project staff. As ProTEST started in the Chawama site prior to the costing, it was not possible to collect full start-up costs there. Start-up costs related to integrating services in Matero were collected, and are presented separately.

Costs were collected retrospectively using a combination of an ingredients-based costing methodology, whereby quantities of resources were multiplied by their respective prices to obtain total costs, and a step-down methodology, where joint costs were allocated to activities using allocation factors [47]. Each service was costed independently, with an allocation of indirect cost for those services provided by the same provider, specifically ProTEST coordination, IPT, Outreach, and ProTEST clinic, which were provided by the ZAMBART project team and the VCT centres which were provided by KCTT. Costs from the ZAMBART project office, that could not be directly allocated to components, including capital costs, were allocated across core services (except VCT, which was provided by KCTT) using direct financial component costs as allocation factor and placed in the category 'Indirect costs'. To allocate KCTT central/administrative costs to the VCT centres and hospice, costs were first allocated to VCT and 'Other services' according to direct costs, then allocated to Chawama and Matero according to the respective sites' proportion of total people tested for HIV. The 'indirect costs' category included depreciation of capital goods, including vehicles which could not be separated out precisely. The hospice was allocated a portion of 'Other' KCTT administration costs according to its share of direct costs in 'Other' direct costs. Because HBC did not record site specific costs, programme costs were allocated proportionately to Chawama and Matero by their share of total patients.

### Outcomes and average costs

Component total costs were divided by the component outcomes to obtain component average costs (unit costs). As the first point of contact with the ProTEST Initiative was through the VCT service, the number of people pre-test counselled was taken as the number of people reached by ProTEST. The impact of VCT was measured using the number of people: pre-test counselled, tested, tested HIV-positive, and completing the VCT process (receiving their results). IPT compliance is known to be a problem [48], therefore both the numbers of people starting and completing IPT were used. People were considered to have completed IPT if they finished the 6-month course within eight months. The indicator used for HBC was the annual number of patients, estimated as the average of the number of patients at the start and at the end of the year. Cost per visit would have been desirable, but was not available. For hospice it was possible to calculate both the cost per admission and the cost per bednight.

### Sensitivity analysis

A univariate sensitivity analysis was performed to understand how assumptions made in this analysis affected the unit costs of ProTEST. We varied the discount rate (0% and 6%), we estimated the impact of full VCT compliance, a twice as rapid loss to follow-up for IPT, and variance in the number of ProTEST clinic visits (up and down by 25%), the allocation factor of ZAMBART joint costs (equal amounts across components, rather than proportionate to direct costs). In addition, the impact of having a international director was tested by replacing the international salary with a local salary, taken as that of the local project coordinator.

## Results

### ProTEST total costs

**Figure 2:**
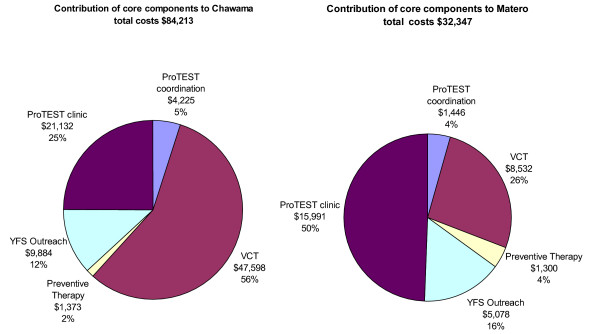
Contribution of core components to site total costs.

Component costs are presented by input in Tables 1, 2, 3, and their contribution to total annual costs in Figure 2. The total annual economic cost of ProTEST core components was $84,213 in Chawama and $32,347 in Matero. The annual economic cost of ProTEST co-ordination was $1,446 in Matero and $4,225 in Chawama, 4%–5% of site costs in both. Matero start-up costs were treated as capital and were annualised using a 3% discount rate, this came to a cost of $899 per year, which would increase ProTEST co-ordination costs to 8% of site costs.

**Table 1:** Total annual cost by component and cost category – Core components in Chawama

	CORE COMPONENTS Chawama				
	
Cost Category	Coordination	VCT	IPT	YFS outreach	Clinic
	ECONOMIC COSTS				
	$	$	$	$	$
**Capital**					
Buildings	0	2147	0	1038	201
Equipment	0	467	0	40	23
Vehicles	44	0	0	0	271
Opening ceremony	349	0	0	0	0
Start-up	0	0	224	0	0
Training	0	639	0	0	0
**Total capital costs**	**394**	**3252**	**224**	**1078**	**495**
**Recurrent**					
Personnel	1791	19169	341	4991	5658
Supplies	276	12835	100	357	5248
Vehicle operating & maintenance	13	2722	0	0	56
Building operating & maintenance	0	1405	0	0	0
Training/workshops	0	292	0	0	86
Outreach	0	1666	0	0	0
Indirect costs	1752	6151	619	3455	9574
Other	0	105	88	3	14
**Total recurrent costs**	**3832**	**44346**	**1149**	**8806**	**20637**

**Component total (economic)**	**4,225**	**47,598**	**1,373**	**9,884**	**21,132**
**Site total (economic)**					**84,213**
	FINANCIAL COSTS				
Component total (financial)	3,600	31,765	1,273	7,100	19,676

Cost represented in 2007 US$

Abbreviations: VCT: voluntary counselling and testing; IPT: isoniazid preventive therapy; YFS: youth friendly services.

**Table 2:** Total annual cost by component and cost category – Core components in Matero

	CORE COMPONENTS Matero				
	
Cost Category	Coordination	VCT	IPT	YFS outreach	Clinic
	ECONOMIC COSTS				
	$	$	$	$	$
**Capital**					
Buildings	0	399	0	340	455
Equipment	0	212	0	243	27
Vehicles	12	0	0	0	228
Opening ceremony	86	0	0	0	0
Start-up	899	0	168	0	0
Training	0	0	0	0	0
**Total capital costs**	**97**	**611**	**168**	**583**	**710**
(including start-up)	996	0	0	0	0
**Recurrent**					
Personnel	547	2627	451	2719	5064
Supplies	162	3368	47	202	3081
Vehicle operating & maintenance	3	424	0	0	47
Building operating & maintenance	0	219	0	0	0
Training/workshops	0	46	0	0	0
Outreach	0	260	0	0	0
Indirect costs	636	959	589	1574	7089
Other	0	18	44	0	0
**Total recurrent costs**	**1349**	**7921**	**1132**	**4495**	**15281**

**Component total (economic)**	**1,446**	**8,532**	**1,300**	**5,078**	**15,991**
(including start-up)	2540				
**Site total (economic)**					**32,347**
	FINANCIAL COSTS				
Component total (financial)	1,308	3,878	1,211	2,353	14,568

Cost represented in 2007 US$

Abbreviations: VCT: voluntary counselling and testing; IPT: isoniazid preventive therapy; YFS: youth friendly services.

**Table 3:** Total annual cost by component and cost category – affiliated services

	AFFILIATED SERVICES		
	Chawama	Matero	Both sites
	
Cost Category	HBC	HBC	Hospice
	ECONOMIC COSTS		
	$	$	$
**Capital**			
Buildings	733	279	56126
Equipment	364	139	3801
Vehicles	545	208	5859
Opening ceremony	0	0	0
Start-up	0	0	0
Training	2010	766	1964
**Total capital costs**	**3652**	**1392**	**67749**
**Recurrent**			
Personnel	9495	3620	40895
Supplies	45055	17177	23437
Vehicle operating & maintenance	1049	400	6382
Building operating & maintenance	265	101	2191
Training/workshops	25	9	1123
Outreach	0	0	0
Indirect costs	0	0	12542
Other	0	0	4774
**Total recurrent costs**	**55890**	**21308**	**91343**

**Component total (economic)**	**59,541**	**22,700**	**159,093**
			
	FINANCIAL COSTS		
Component total (financial)	13,483	5,140	60,739

Cost represented in 2007 US$

Abbreviations: HBC: home based care.

Total annual economic costs of VCT were $47,598 in Chawama and $8,532 in Matero, of which 27% and 39% were for supplies. Financial costs for supplies were 30% and 18% of their economic costs, in Chawama and Matero, respectively, primarily due to large donations of HIV test kits. The total annual incremental cost of adding IPT on to VCT was $1,373 in Chawama and $1,300 in Matero, where x-rays were a routine part of the screening process.

Outreach by YFS was $9,884 and $5,078 in Chawama and Matero per year, respectively. In Chawama, the higher outreach costs were largely due to a 4-month outreach intervention targeted at sex-workers and their clients. Much of the outreach by YFS was run by volunteers, leading to the economic costs being more than twice the financial costs in the new site. The annual cost of the clinic was $21,132 and $15,991 in Chawama and Matero, respectively, which contributed to 25% and 49% of total costs in each, respectively.

There was a large difference between the financial and economic costs in the affiliated services, HBC and hospice, with their economic costs being 4.4 and 2.6 times their financial costs. HBC paid for only 39% of labour and 13% of supplies as their care-givers were primarily volunteers and they received large food donations from international organisations such as the World Food Programme. The hospice received 85% of their supplies in donations of which 57% was medicines supplied by the Archdiocese of Lusaka and 43% was other supplies coming from community donations. A further breakdown of personnel and supply inputs and their contribution to personnel and supplies, respectively, can be found in Table 4. No breakdown is given where supplies were less than 10% of total economic costs.

**Table 4:** Major inputs contributors (>10%) in the largest economic cost categories (Personnel and Supplies)

	Personnel Inputs	% of personnel economic costs		Supplies Inputs	% of supplies economic costs	
		Chawama	Matero		Chawama	Matero
Coordination	Director	35%	45%	<10% of total economic costs		
	Project coordinator	32%	27%			
	Meeting attendees	21%	10%			
VCT	Counsellors	64%	94%	Test kits	43%	44%
	Mobilisers	18%	N.A.	Information pamphlets	24%	14%
				Office supplies	7%	27%
IPT	Counsellors	45%	63%	Isoniazid tablets	13%	7%
YFS outreach	YFS volunteers	58%	80%	<10% of total economic costs		
Clinic	Medical doctor (director)	13%	17%	Drugs & medical supplies	99%	98%
	Medical doctor (project coordinator)	61%	58%			
	Medical officer	16%	16%			
	Driver	10%	9%			
		Both sites			Both sites	
HBC	Core staff	16%		Food	89%	
	Urban volunteer	70%				
Hospice	Care givers (paid, volunteer)	(19%, 9%)		Drugs & medical supplies	49%	
	Nurses	10%		Community donations (mostly food)	36%	

VCT: voluntary counselling and testing; IPT: isoniazid preventive therapy; YFS: youth friendly services; HBC: home based care.

### Outcomes

Client flows through the various ProTEST components are presented in Table 5. The number of people reached by ProTEST was 3,604 in Chawama and 258 in Matero (7% of the mature site) during the year of the costing. Coordination meetings were attended on average by 35 people in Chawama and 21 people in Matero. Almost half the people pre-test counselled were tested in Chawama, whereas all people were tested in Matero. Around 90% (88%–93%) of people tested completed the VCT process in both sites. There was a large disparity between the HIV prevalence rates among VCT clients in the sites, 64% in Matero and 29% in Chawama. Compliance with preventive therapy in Matero (47%) was twice as high as compliance in Chawama (23%). There were an estimated 1,020 visits to ProTEST clinic in Chawama and 638 visits in Matero. Hospice had 288 admissions. HBC cared for 400 and 153 patients in Chawama and Matero, respectively.

**Table 5:** Outputs and unit costs

	**Chawama**		**Matero**	
	Outputs	Unit cost	Outputs	Unit cost
		
**Core components**				
ProTEST coordination				
person reached	3,604	$1	258	$6
(including start-up)				($9)
VCT				
people pre-test counselled	3,604	$13	258	$33
people tested	1,575	$30	258	$33
people receiving results	1,381	$34	239	$36
HIV+ cases found	455	$105	166	$51
IPT				
people starting	204	$7	73	$18
people completing	47	$29	35	$38
ProTEST clinic				
clinic visits	2115	$10	638	$25
clients eligible for clinic*	399	$53	154	$104
**Affiliated services**				
HBC**				
clients	400	$149	153	$149
Hospice***				
admissions	288	$552		
bednights	6685	$24		

Cost represented in 2007 US$

Abbreviations: VCT: voluntary counselling and testing; IPT: isoniazid preventive therapy; HBC: home based care.

* Estimated number of clients attending clinic, based on those eligible: HIV+ and collected results.

** Costing for Jan-Dec 2000

*** hospice was in one location only, but receive patients from the surrounding areas, including Matero and Chawama

### Average costs

The annual cost of ProTEST co-ordination was $1 and $5 per person reached in Chawama and Matero, respectively (Table 5). The VCT cost per person pre-test counselled was $13 in Chawama and $33 in Matero. Due to the high attrition rate between pre-test counselling and testing, the cost per person tested and cost per person completing the VCT process did not vary as much between the sites. The cost per person completing IPT ($29) was over four times the cost per person starting IPT ($7) in Chawama. In Matero it was about double, $38 and $18, respectively. The cost per clinic visit was $10 in Chawama and $25 in Matero. The estimated number of visits per patient was 2.6 and 4.1 in Chawama and Matero, respectively. This leads the annual cost per client in Matero ($104) to be about double that in Chawama ($53).

### Sensitivity analysis

The impact of a number of assumptions made were explored for their impact on unit costs (Tables 6 and 7). The choice of allocation factor for ZAMBART office costs had the greatest impact on component costs. When allocated equally across components and sites, total Matero costs increased. In both sites unit costs for IPT increased by about 3-fold, while ProTEST clinic unit costs dropped by 30% and 25% in Chawama and Matero, respectively. Variations in discount rate, and a faster IPT drop-out rate had a small impact on unit costs, less than 10% variation in unit costs. Had full VCT compliance been assumed, rather than actually measured, we would have dramatically underestimated the cost per person tested, and per person completing the full VCT process. Although the costs were fairly robust to assumptions made in this analysis, they were quite sensitive to the allocation factors used for the indirect costs from ZAMBART, and the number of clinic visits. We also modelled the impact of the ProTEST Initiative without an international director (Table 8). This would reduce the costs of coordination by about a quarter.

**Table 6:** Sensitivity analysis: The impact of discount rate on unit costs (in percentage change)

	Baseline Unit costs Discount rate 3%		Discount rate 0%		Discount rate 6%	
Cost per	Chawama	Matero	Chawama	Matero	Chawama	Matero
ProTEST coordination						
person reached	$1.10	$5.26	-1%	-1%	1%	1%
VCT						
people pre-test counselled	$12.40	$31.05	-0.2%	-0.3%	0.2%	0.3%
people tested	$28.38	$31.05	-0.2%	-0.3%	0.2%	0.3%
people receiving results	$32.36	$33.52	-0.2%	-0.2%	0.2%	0.3%
HIV+ cases found	$98.22	$48.26	-0.2%	-0.2%	0.2%	0.2%
IPT						
people starting	$6.32	$16.71	0%	0%	0%	0%
people completing	$27.48	$35.29	0%	0%	0%	0%
						
ProTEST clinic						
clinic visits	$9.38	$23.53	-0.3%	-0.3%	0.3%	0.3%
clients eligible for clinic	$49.73	$97.64	-0.3%	-0.3%	0.3%	0.3%
						
HBC						
clients	$139.77	$139.77	-0.2%	-0.2%	0.2%	0.2%
Hospice						
admissions	$518.68		-0.4%		0.4%	
bednights	$22.35		-0.4%		0.4%	

Cost represented in 2007 US$

Abbreviations: VCT: voluntary counselling and testing; IPT: isoniazid preventive therapy; YFS: youth friendly services; HBC: home based care.

**Table 7:** Sensitivity analysis: The impact of equal allocation of joint ZAMBART costs and divers assumptions on unit costs (in percentage change)

	Baseline Unit costs		Equal allocation of joint ZAMBART costs*		Diverse assumptions			
Cost per	Chawama	Matero	Chawama	Matero	Chawama		Matero	
ProTEST coordination								
person reached	$1.10	$5.26	34%	175%				
VCT					Full VCT compliance			
people pre-test counselled	$12.40	$31.05	no change to baseline		16%		151%	
people tested	$28.38	$31.05	no change to baseline		-49%		10%	
people receiving results	$32.36	$33.52	no change to baseline		-56%		-4%	
HIV+ cases found	$98.22	$48.26	no change to baseline		-56%		-54%	
IPT					PT fast drop off of loss to follow-up			
people starting	$6.32	$16.71	185%	198%	-8%		160%	
people completing	$27.48	$35.29	185%	198%	-8%		26%	
					Estimated number of visits			
ProTEST clinic					25% ↑	25% ↓	25% ↑	25% ↓
clinic visits	$9.38	$23.53	-30%	-25%	-20%	33%	101%	235%
clients eligible for clinic	$49.73	$97.64	-30%	-25%				
								
HBC								
clients	$139.77	$139.77	no change to baseline					
Hospice			no change to baseline					
admissions	$518.68		no change to baseline					
bednights	$22.35							

Cost represented in 2007 US$

Abbreviations: VCT: voluntary counselling and testing; IPT: isoniazid preventive therapy; YFS: youth friendly services; HBC: home based care.

* to the service provided by ZAMBART: coordination, IPT, YFS, ProTEST clinic

**Table 8:** The impact of no international staff on unit costs (in percentage change)

	Baseline Unit costs		No international staff**	
Cost per	Chawama	Matero	Chawama	Matero
ProTEST coordination				
person reached	$1.10	$5.26	-23%	-26%
VCT				
people pre-test counselled	$12.40	$31.05	no change to baseline	
people tested	$28.38	$31.05	no change to baseline	
people receiving results	$32.36	$33.52	no change to baseline	
HIV+ cases found	$98.22	$48.26	no change to baseline	
IPT				
people starting	$6.32	$16.71	-5%	-5%
people completing	$27.48	$35.29	-5%	-5%
				
ProTEST clinic				
clinic visits	$9.38	$23.53	-9%	-11%
clients eligible for clinic	$49.73	$97.64	-9%	-11%
				
HBC				
clients	$139.77	$139.77	no change to baseline	
Hospice				
admissions	$518.68		-1%	
bednights	$22.35		-1%	

** The salary of the international director was replaced with that of the project coordinator.

Cost represented in 2007 US$

Abbreviations: VCT: voluntary counselling and testing; IPT: isoniazid preventive therapy; YFS: youth friendly services; HBC: home based care.

## Discussion

This study looked at the costs of providing a package of care to PLWH as implemented in the Zambian ProTEST Initiative. It not only looks at service components (VCT, IPT, clinic, outreach, and hospice and HBC) but also measures the cost of co-ordinating these services to provide a core package of care for PLWH. Annual costs of the core package of services ranged from $32,347 in the smaller site to $84,213 in the larger site. The introduction of ProTEST has led to enhanced collaboration and improved referral systems for as little as $1 per person reached.

There were a number of striking differences between the two sites. The Matero site operated on a much smaller scale than the Chawama site. The client numbers were mostly constrained on the supply-side. Group counselling in Chawama made it possible to reach many people with pre-test counselling. The Matero VCT service had no space for group counselling, there was only a single counsellor, and the health centre (including the VCT service) was closed for a couple of months. On the demand side, it takes time for people to hear about the service and start coming. When people do start presenting, new VCTs tend to attract the people with more advanced symptoms of AIDS [49,50].

The HIV prevalence in Matero was very high (64%), while the HIV prevalence among VCT clients in Chawama (29%) was more representative of the prevalence in the general population (22%). This difference can also be recognised in the behaviour of clients. While in Matero a very high proportion of clients completed the full process (92%), the Chawama VCT experienced high rates of people not proceeding to testing after pre-test counselling. The high drop-out rate may have a few reasons. Group counselling made it possible to pre-test counsel large groups. Many group counselling sessions were for school children who needed parental permission to test. These youths were educated about HIV at Chawama, but unable to test. Additionally, mobilisers received a bonus for each person presenting for pre-test counselling with their referral slips. The number of ProTEST clinic visits per client in Matero was 62% higher than Chawama again suggesting a more advanced stage of HIV in the clients in Matero.

### Limitations

A number of caveats must be considered in this analysis. The allocation of indirect costs had a large influence on component costs, although not on costs of ProTEST as a whole. As start-up costs were not collected for Chawama, both site costs are presented excluding start-up costs. A number of variables were estimated retrospectively, such as staff time allocations. The line item of HIV test kits for VCT was modelled as KCTT had complemented their purchased test kits with donated kits of unknown number. Quantities were estimated by the number of HIV test performed plus an estimated 10% wastage. International prices for these kits were then applied. The HBC costs are the average costs over all sites, and are therefore not site specific. Cost-effectiveness in terms of HIV infections averted would need to be estimated in order to compare ProTEST with other HIV interventions. This requires behavioural surveys and modelling or a randomised intervention design. Although the direct costs of research were removed, time input by international staff into the daily running and co-ordination of the intervention were included at full costs. Therefore if replicating ProTEST outside of the pilot intervention setting cost, personnel cost particularly, are expected about a quarter lower (Table 8).

A more general limitation of this study is that it was conducted before the widespread availability of ART. The integration of services is however as relevant in the ART era as at the time of this study, but costs due to the provision of ART would need to be factored in.

### Comparison with other studies

The cost of VCT was around $35 per client receiving results, which is consistent with other studies of the cost of VCT in Africa. Sweat et al. estimated the cost of VCT in Kenya and Tanzania and found a cost per VCT client of $34 and $37 (adjusted to 2007 dollars) [29]. A key difference from our study is that Sweat et al. assumed all clients presenting for pre-test counselling return for their test results, whereas we were able to measure this and estimate average costs for multiple VCT outputs, incorporating attrition rates. Aisu et al. estimated the cost of VCT in Uganda to be $30 [37]. Our cost per client tested did not differ greatly from these estimates ($34 and $36). Forsythe estimated the incremental financial cost of VCT integrated into health centres at $19 per client [34]. It is not clear what 'client' means in terms of completing the VCT process. These differences make it difficult to compare these results with our VCT costs. Despite low IPT adherence, its implementation costs were relatively low: $29 to $38 per person completing IPT. The modelled estimate of the cost effectiveness of IPT by Shresta et al. in Uganda was $58 [51]. We have not considered the additional benefits of early detection of active TB or the medical cost averted due to averting TB infections. Three studies modelled the treatment cost savings attributable to IPT in Africa using secondary data [30,33,36]. Bell estimated a savings in terms of medical costs averted of $34 per person [36]. Masobe et al. performed a least cost analysis for a hypothetical cohort of 100,000 HIV-positive patients over 8 years and estimated IPT could result in a net savings of $19 million (or $193 per person) [30]. Foster et al. included in benefits not only treatment costs, but also lost wages averted and found a benefit cost ratio of 1.86 [33].

The larger number of annual clinic visits per patient to ProTEST clinic in Matero (4.1) than in Chawama (2.6) is consistent with the findings by Kinghorn et al. who found the annual number of outpatient visits in South Africa increased strongly from stage 1 HIV (3.8 visits annually) to stage 4 HIV (6.29 visits annually) [52]. The cost per outpatient hospital visit for PLWH in Thailand was $27 [53], again very close to the cost per ProTEST clinic visit. The daily cost of hospice care is shown to be within the range of in-patient hospital cost for AIDS patients in low-income African countries, ranging from $9 to $49. Hansen et al. estimate the cost of HBC in Zimbabwe ranging from $23 to $33 per visit in two urban areas, this came to $34–$253 per patient per year, with a large difference between the sites in the average number of visits per patient [32].

## Conclusion

In many locations a range of preventive and clinical services for HIV exist. This study shows that it is feasible to integrate these services to provide a package of care for PLWH. Without such a continuum of care ART provision makes no sense. VCT must be expanded to allow access to ART. Some clients may not yet need ART but still need screening for other diseases, such as TB, and provision of preventive therapy. Patients accessing ART also need screening for TB and may similarly still require preventive therapies, although the additional benefit of preventive therapy in this setting is currently unclear. The provision of ART still requires a broader range of services for PLWH including symptom-; symptom management, palliative care and HBC will still be required by some patients. Without integration of services and collaboration between service providers, HIV care will not be successful.

## List of abbreviations

ART Anti-retroviral therapy

HBC Home based care

IPT Isoniazid preventive therapy

PLWH People living with HIV

TB Tuberculosis

VCT Voluntary counselling and HIV testing

YFS Youth friendly services

## Competing interests

The author(s) declare that they have no competing interests.

## Authors' contributions

All authors read and approved the manuscript. FTP conducted the cost analysis and drafted the paper. LK designed and supervised the cost analysis and participated in writing the paper. RG ran the project, assisted in collecting cost data, participated in the interpretation of the results and helped to draft the paper. HA supervised the project and assisted in writing the paper. IK collected the costs for the VCT and assisted in the cost analysis of the VCT. MH collected the costs of HBC and assisted in the interpretation of the results. PGF assisted in the overall interpretation of the study results and writing the paper.
